# Elimination of Deoxynivalenol, Aflatoxin B1, and Zearalenone by Gram-Positive Microbes (*Firmicutes*)

**DOI:** 10.3390/toxins14090591

**Published:** 2022-08-27

**Authors:** Cintia Adácsi, Szilvia Kovács, István Pócsi, Tünde Pusztahelyi

**Affiliations:** 1 Doctoral School of Nutrition and Food Sciences, University of Debrecen, Böszörményi Str. 138, H-4032 Debrecen, Hungary; 2 Central Laboratory of Agricultural and Food Products, Faculty of Agricultural and Food Sciences and Environmental Management, University of Debrecen, Böszörményi Str. 138, H-4032 Debrecen, Hungary; 3 Department of Molecular Biotechnology and Microbiology, Institute of Biotechnology, Faculty of Science and Technology, University of Debrecen, Egyetem Tér 1, H-4032 Debrecen, Hungary

**Keywords:** mycotoxins, cell wall, peptidoglycan, S-layer, zearalenone, elimination, esterase

## Abstract

Mycotoxin contaminations in the feed and food chain are common. Either directly or indirectly, mycotoxins enter the human body through the consumption of food of plant and animal origin. Bacteria with a high mycotoxin elimination capability can reduce mycotoxin contamination in feed and food. Four Gram-positive endospore-forming bacteria (*Bacillus thuringiensis* AMK10/1, *Lysinibacillus boronitolerans* AMK9/1, *Lysinibacillus fusiformis* AMK10/2, and *Rummeliibacillus suwonensis* AMK9/2) were isolated from fermented forages and tested for their deoxynivalenol (DON), aflatoxin B1 (AFB1), and zearalenone (ZEA) elimination potentials. Notably, the contribution of bacterial cell wall fractions to the observed outstanding ZEA elimination rates was demonstrated; however, the ZEA elimination differed considerably within the tested group of Gram-positive bacteria. It is worth noting that the purified cell wall of *L. boronitolerans* AMK9/1, *L. fusiformis* AMK10/2 and *B. thuringiensis* AMK10/1 were highly efficient in eliminating ZEA and the teichoic acid fractions of *B. thuringiensis* AMK10/1, and *L. fusiformis* AMK10/2 could also be successfully used in ZEA binding. The ZEA elimination capacity of viable *R. suwonensis* AMK9/2 cells was outstanding (40%). Meanwhile, *R. suwonensis* AMK9/2 and *L. boronitolerans* AMK9/1 cells produced significant esterase activities, and ZEA elimination of the cell wall fractions of that species did not correlate with esterase activity. DON and AFB1 binding capabilities of the tested bacterial cells and their cell wall fractions were low, except for *B. thuringiensis* AMK10/1, where the observed high 64% AFB1 elimination could be linked to the surface layer (S-layer) fraction of the cell wall.

## 1. Introduction

Mycotoxin production by molds is a global problem that cannot be solved easily in the field, even with the introduction of highly efficient proper agricultural practices. Environmental conditions affected by climate change and improper storage conditions may facilitate mold growth and secondary metabolite production [1,2]. These fungi can infect many plants and a wide array of food and agricultural products, and they can also produce a broad spectrum of harmful secondary metabolites, such as carcinogenic aflatoxins (AFs) [3,4]. Another dangerous mycotoxin, zearalenone (ZEA), is produced mainly by *Fusarium* species [5], and due to its estrogenic character, ZEA and its derivatives may cause severe reproductive and sexual dysfunctions [6,7,8]. The co-occurrence of ZEA with another *Fusarium* mycotoxin deoxynivalenol (DON) is common [9,10], and DON is a potent inhibitor of the protein synthesis in *Eukarya* [11], causing nausea, vomiting, diarrhoea, or anorexia in animals [12].

Not surprisingly, removing these harmful mycotoxins from feed and food is a crucial issue worldwide. In addition to the extensive range of available physical and chemical mycotoxin decontamination methods, biological technologies often based on food-grade microorganisms are gaining ground. The tested biological detoxification methodologies typically incorporate biodegradation and biosorption processes [13,14,15,16]. Mycotoxin elimination by bacteria and yeasts can happen through binding to biopolymers (e.g., peptidoglycan, PG), degradation by enzymes (e.g., esterase), or enzymatic conjugation to various molecules, giving rise to so-called masked or bound mycotoxins that make the original mycotoxin forms hidden to classic chemical analytical methods [17].

Bacteria are distinguished tools in the biological control of mycotoxigenic molds and the mycotoxins themselves. For example, bacterial cells can adsorb mycotoxins, e.g., aflatoxins are bound effectively by various cell wall components [18], or can detoxify these contaminants via enzymic degradation [15,19], or can inhibit the growth of molds by different antifungal metabolites, e.g., bacteriocins and organic acids [20].

Gram-positive lactic acid bacteria (LAB) typically have an excellent mycotoxin elimination capability, and both viable and nonviable LAB can eliminate aflatoxin B1 (AFB1). However, the elimination depends on the genus, pH, and bacterial density [21]. The binding of some major mycotoxins, including AFB1 [22], ZEA [23], and certain trichothecenes [24] by some probiotic LAB, was also demonstrated in vitro. Furthermore, some non-lactic acid bacteria, including *Bacillus* spp., were also investigated for their possible biotechnological application in mycotoxin elimination [18].

**Figure 1:**
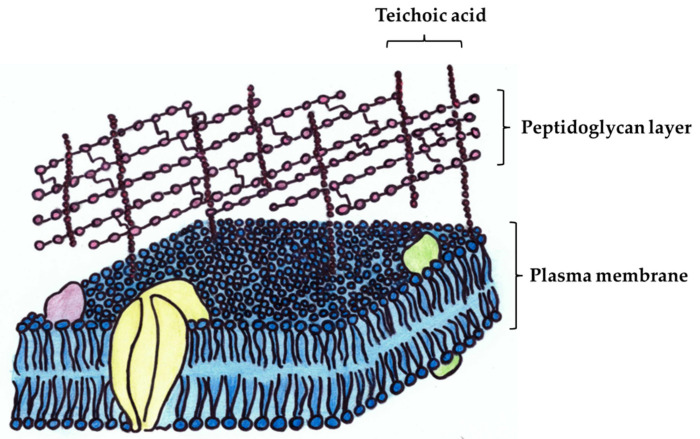
Main structural elements of the Gram-positive cell wall. The schematic figure did not show S-layer proteins present in the outermost part of the cell wall. Besides the main structural similarities, at the microstructural level, microbial cell walls are diverse and, therefore, highly differ in mycotoxin elimination capabilities.

The bacterial cell wall consists of several biopolymers that can absorb different toxic substances, such as mycotoxins. Therefore, differences between Gram-negative and Gram-positive bacterial cell walls (Figure 1) would allow for predicting different adsorption capabilities in mycotoxin elimination due to the apparent differences in the thickness of the PG layer and the presence of the outer membrane. The surface layer, which is the outermost cell wall layer in many *Bacteria* and *Archaea* cells, attaches to the PG by electrostatic interactions and possesses inherent, entropy-driven affinities to self-assemble with each other [25] or, in the Gram-negative bacterial cell wall, to attach to outer membrane lipopolysaccharides (LPS) [26].

In this work, bacteria and bacterial cell wall preparations were screened for mycotoxin elimination using a unique collection of endospore-forming Gram-positive bacteria isolated from forages in Hungary. Novel mycotoxin-eliminating bacteria were aimed to find and shed light on cell wall fractions and other cell constituents, such as enzymes, which have the most critical role in mycotoxin elimination.

## 2. Results

### 2.1. Identification of Isolated Bacteria

Bacterial isolates from fermented forages were identified with higher than 97% homology using 16S rRNA gene sequences (Table 1). All bacteria were Gram-positive endospore-forming organisms and taxonomically belonged to the phylum *Firmicutes*. Except for *Rummeliibacillus suwonensis* (*Planococcaceae*), all isolates were placed in the family *Bacillaceae.*

### 2.2. Mycotoxin Elimination

Viable cells of *R. suwonensis* AMK9/2, *L. boronitolerans* AMK9/1, *L. fusiformis* AMK10/2, and *B. thuringiensis* AMK10/1, as well as their cell wall fractions, were supplemented with zearalenone (ZEA), aflatoxin B1 (AFB1), and deoxynivalenol (DON), respectively, to study the mycotoxin elimination capability.

**Figure 2:**
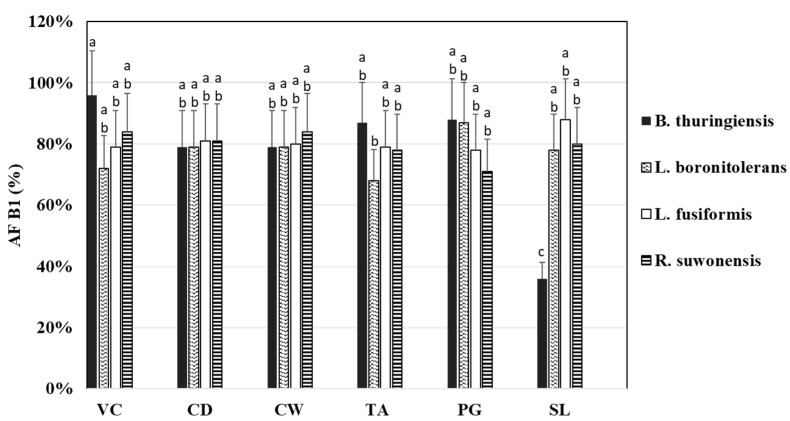
Aflatoxin B1 (AFB1) measured from the supernatants after elimination test with viable cells (*Bacillus thuringiensis* AMK10/1, *Lysinibacillus boronitolerans* AMK9/1, *Lysinibacillus fusiformis* AMK10/2, and *Rummeliibacillus suwonensis* AMK9/2) and their cell wall fractions. Viable cells (VC), cell debris (CD), purified cell wall (CW), teichoic acid fraction (TA), peptidoglycan fraction (PG), and S-layer fraction (SL) were tested. The letters above each column indicate the results of pairwise comparisons of all samples. Results that share the same letter do not differ significantly (*p* ≤ 0.05) from one another in AFB1 elimination.

All samples showed negligible DON elimination under the tested experimental conditions. Furthermore, AFB1 elimination rates were low and typically under 20% by all tested bacteria and their cell wall fractions except the S-layer fraction of *B. thuringiensis* AMK10/1, which eliminated 64% of AFB1 (Figure 2).

**Figure 3:**
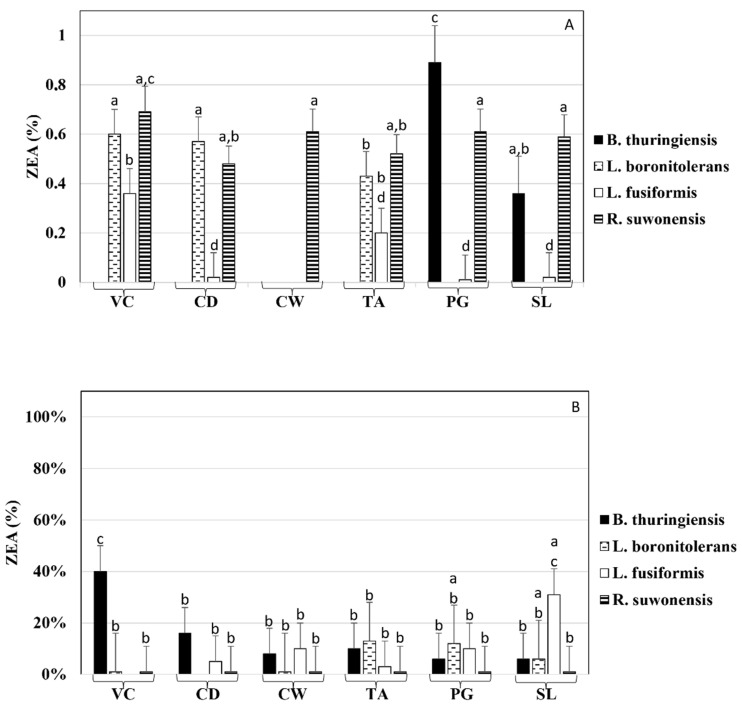
Zearalenone (ZEA) was measured from the supernatant (**A**) and the extract of pellets (**B**) after the elimination test with viable cells (*Bacillus thuringiensis* AMK10/1, *Lysinibacillus boronitolerans* AMK9/1, *Lysinibacillus fusiformis* AMK10/2, and *Rummeliibacillus suwonensis* AMK9/2) and their cell wall fractions. Viable cells (VC), cell debris (CD), purified cell wall (CW), teichoic acid fraction (TA), peptidoglycan fraction (PG), and S-layer fraction (SL) were tested. The letters above each column indicate the results of pairwise comparisons of all samples. Bars that share the same letter do not differ significantly (*p* ≤ 0.05) from one another in ZEA content.

The elimination of ZEA by the bacteria and their cell wall preparations showed remarkable species-specific differences (Figure 3). In many cases, bacterial cells and their cell wall preparations showed remarkable ZEA elimination rates (Figure 3A), but the eliminated ZEA could only be partially recovered from the bacteria and cell wall pellets (Figure 3B). In addition, for some bacteria and cell wall preparations, the quantity of the recovered ZEA remained even below the detection limit (LOD, 2.6 µg/L).

Interestingly, ZEA elimination by viable *R. suwonensis* AMK9/2 cultures and its cell wall preparations showed approximately the same values: about 40% (Figure 3A). Significantly, purified cell wall, PG, and S-layer protein fractions of *L. boronitolerans* AMK 9/1 nearly eliminated ZEA, and the remaining ZEA concentrations in the supernatants were below LOD. ZEA concentrations recovered from the various cell wall fraction pellets were 1–12% of the starting values (cell wall fraction: 1%, PG: 12%, S-layer proteins: 6%) (Figure 3B).

*L. fusiformis* AMK10/2 cell debris, purified cell wall, PG fraction, and S-layer protein fraction were valuable for eliminating ZEA (Figure 3A). However, only small portions of the eliminated ZEA could be recovered from the fractions, except for the S-layer fraction of AMK10/2, where 25% of the original ZEA could be extracted (Figure 3B).

Considering *B. thuringiensis* AMK10/1, viable cells, cell debris, purified cell wall, and teichoic acid fractions were suitable for efficiently eliminating ZEA from the liquid phase (Figure 3A). Interestingly, the elimination of ZEA by PG fraction of the AMK10/1 strain was almost negligible, while, for the S-layer fraction, it was about 38%. Meanwhile, the teichoic acid fraction was the most important cell wall fraction for ZEA elimination (Figure 3A). Nevertheless, AMK10/1 cells and cell wall fractions released ZEA under extraction (Figure 3B) and the most significant release was achieved from the total cells.

### 2.3. Esterase Activity

**Figure 4:**
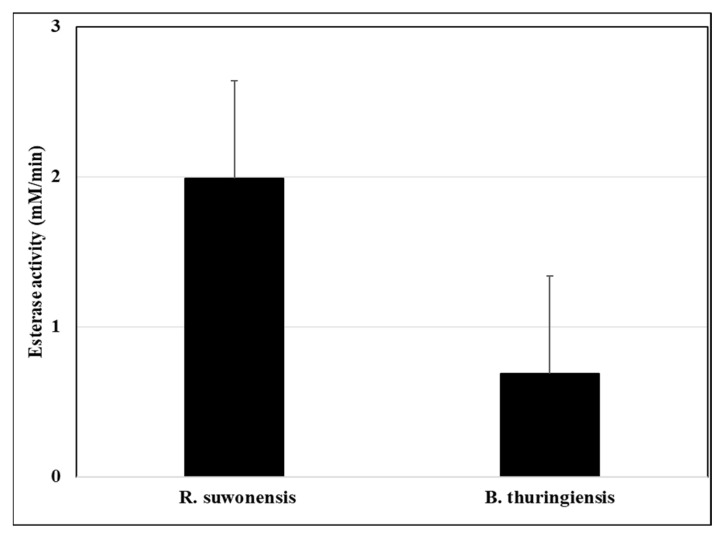
Esterase activity was measured from the lysed cell debris of *Rummeliibacillus suwonensis* AMK9/2 and *Bacillus thuringiensis* AMK10/1.

Since esterase activity is connected to ZEA degradation in living bacterial cells [19], this enzyme activity was measured and varied in a wide range. *R. suwonensis* AMK9/2 and *L. boronitolerans* AMK9/1 produced high esterase activities compared to *L. fusiformis* AMK10/2 and *B. thuringiensis* AMK10/1, the highest enzyme activity was measured in *R. suwonensis* AMK9/2 cell debris (1.98 ± 0.3 mM *p*-nitrophenol released/min) (Figure 4). Correlation analysis revealed a correlation coefficient of 0.618 between enzyme activity and extracted ZEA from cell debris and −0.748 between enzyme activity and the ZEA remaining in the cells’ supernatant. AMK 9/2 cells and AMK 9/2 cell wall fractions eliminated ZEA with the same ratio. However, esterase activity could not be measured from the cell wall fractions because those samples were produced using different chemicals and heat treatment. Therefore, esterase activity could not explain ZEA elimination by AMK 9/2 cell wall fractions.

## 3. Discussion

Mycotoxin elimination technologies can use viable and dead bacterial biomasses and various cell fractions. The mycotoxin adsorption capability of bacteria depends primarily on cell wall components [23,24], where mainly polysaccharides, such as peptidoglycan (PG) and teichoic acids, play a critical role in mycotoxin binding [31]. Besides adsorption, enzymatic degradation (e.g., [14]) is possible, making a cell-free biotechnological process for mycotoxin elimination achievable.

Elimination of zearalenone (ZEA), deoxynivalenol (DON), and aflatoxin B1 (AFB1) using forage-based isolates of the Gram-positive *R. suwonensis, L. boronitolerans, L. fusiformis,* and *B. thuringiensis* was targeted. For *R. suwonensis* AMK9/2 and *L. boronitolerans* AMK9/1, no mycotoxin elimination-related studies are published thus far, to the best of our knowledge. For *L. fusiformis*, there was only one study on AFB1 elimination [32] but it was only performed with viable bacteria and not with cell fractions; meanwhile, for *B. thuringiensis,* there are several papers available on its antagonistic effects on patulin-producing fungi and its lactonase production, which degrades patulin mycotoxin (e.g., [33]). In the present study, viable cells, cell wall debris, purified cell walls, teichoic acid, PG, and S-layer fractions were produced with heat and different chemical treatments and tested in mycotoxin elimination experiments.

Both heat and organic acids affect the adsorption of mycotoxins by the cell walls because both factors reduce the PG’s thickness, increasing the pore size of the structure [22,23]. In addition, heat treatment disrupts the glycosidic bonds of cell wall polysaccharides, such as PG, and organic acids break the amide ligaments in the structure of PG [22,23]. These treatments also denature proteins and split them into smaller peptides by breaking peptide bonds and exposing more binding sites [34]. Protein denaturation caused by high temperature is also a part of pore generation, resulting in enhanced permeability in living cells [35].

Under the experimental conditions, the trichothecene mycotoxin DON was not eliminated by bacterial cells and cell fractions, unlike in other Gram-positive cells [21,36,37]. Success in controlling DON-producing fungi by microbial antagonists and detoxifying DON with microbes and enzymes was reported in the past several years [16]. El-Nezami et al. [24] reported on significant differences in the ability of bacteria to bind trichothecenes in vitro. Despite heat and acid treatments, which significantly enhanced the ability of Gram-positive lactic acid bacteria to remove DON from the MRS medium [38], these parameters did not increase DON elimination yields. Results on presented Gram-positive bacteria or cell wall fractions suggest the lack of degrading enzymes or adsorption.

In *Lacticaseibacillus rhamnosus*, aflatoxin binding occurred through the carbohydrate and protein contents of the cell wall [22]. For *B. thuringiensis* AMK10/1, the S-layer fraction had the highest potential in AFB1 elimination. Lahtinen et al. [39] emphasized the significant role of the carbohydrate moieties of PG and other structures closely associated with PG in the binding process. Moreover, the teichoic acid fraction of *B. thuringiensis* showed a more significant AFB1 eliminating potential than the intact bacterial cells [39]. Similar observations were reported by Hernandez-Mendoza et al. [40] for *Lactobacillus reuteri* NRRL14171 and *Lactobacillus casei* Shirota [40], where the teichoic acid-deficient microbes bound significantly lower amounts of AFB1. Other lactic acid bacteria showed that the higher the D-alanine or teichoic acid contents, the higher level of AFB1 bound [41].

Teichoic acids may constitute up to 50% of the bacterial cell wall [42], but its amount is species-specific. Some reports indicated that the extraction of teichoic acids is likely influenced by the extraction time and the applied trichloroacetic acid (TCA) concentration [43]. Therefore, teichoic acid-based elimination of aflatoxins should be carefully interpreted when comparing various bacterium species. Adebo et al. [32] also suggested enzymatic degradation of AFB1 by an extracellular enzyme produced by *L. fusiformis*. However, the enzymatic reactions were carried out at an AFB1 concentration of 2200 ppb (3–24 h incubations), almost two orders of magnitude higher than the AFB1 concentration employed in this study.

Considering the elimination of ZEA, *L. fusiformis* AMK10/2 viable cells eliminated less mycotoxin than dead bacteria, indicating massive, heat-induced changes in the conformation of cell wall biopolymers, increasing the number and availability of ZEA binding sites [23]. Similarly, *L. rhamnosus* cell wall polysaccharide components showed good ZEA adsorption potential, and both heat and acid treatments significantly enhanced the ZEA adsorption capability of the fractions [24]. In addition, ZEA was bound predominantly by carbohydrate components of the cell wall of lactic acid bacteria [44]. Interestingly, the ZEA elimination capability of *R. suwonensis* AMK9/2 was identical by both viable and heat-treated bacterial cells and their fractions. Therefore, we can assume that the applied treatments did not affect cell wall structures to modify ZEA elimination yields.

Enzymatic degradation can also play a role in ZEA elimination. Tinyiro et al. [45] demonstrated that the quantities of ZEA bound by autoclaved and acid-treated cells of a *Bacillus* strain were identical, while suggesting that a metalloenzyme was responsible for ZEA degradation by a *Bacillus natto* strain. Several recent studies supported the view that bacterial esterase activities could contribute to ZEA degradation. For example, Wang et al. [19] selected ZEA-eliminating bacteria based on their esterase activities. In this study, *R. suwonensis* AMK9/2 showed the highest cell wall-bound esterase activity, but the esterase activities did not correlate to the eliminated ZEA in cell wall fractions. It was also demonstrated that even much higher esterase activities did not result in significantly improved ZEA eliminations (14–52%, depending on the strains) under ZEA expositions of *Lactiplantibacillus plantarum* [46]. Esterase activities of ruminal bacteria were also tested for deacetylating *Fusarium* T-2 toxin and showed varying success rates [47]. In *Lactobacillus,* ZEA elimination was suggested to occur through adsorption and not enzymatic degradation [23]. In the case of *B. thuringiensis* AMK10/1, the amounts of ZEA eliminated by the cell fractions were below those eliminated by the viable cells. The most significant ZEA release was also achieved from the viable cells, suggesting adsorption without enzymatic degradation.

## 4. Conclusions

Application of cell wall fractions for mycotoxin elimination can be more successful in some cases compared to viable cells. Cell-free technologies applying only cell fractions have a future in postharvest technologies. Industries can use bioadsorption techniques without producing metabolites with unknown physiological effects. Besides preharvest biocontrol techniques, bioadsorption can be a suitable method for decreasing mycotoxin presence. Species-specific elimination abilities could be originated from the diverse available adsorption surfaces of the microbial cell wall. However, the effect of the chemical treatment (which resulted in new surfaces and binding sites) should also be considered for its positive effect on making more potent microbial bioadsorption matrixes. 

## 5. Materials and Methods

### 5.1. Isolation of Bacteria

Fermented forages were collected at the final stage of the fermentations (after 4–6 weeks) from different Hungarian dairy cattle farms in 2019–2020. The producers collected ten parallel samples per site from freshly opened silos and bales, combined them in sterile Velcro bags (min. 5 kg), and transferred them to the analytical laboratory for further analysis [48].

Fermented forage samples (100 g) placed in sterile homogenizing Stomacher bags were suspended in a 1:9 ratio buffered peptone water (BPW) solution (Scharlab, Barcelona, Spain) and homogenized with a Stomacher masticator homogenizer (IUL Instruments, Barcelona, Spain). Following that, decimal dilutions were made of the suspensions, and total microbial counts were determined on plate count agar (Scharlab, Barcelona, Spain) medium applying the pour plate method. Inoculated solid agar media were incubated at 30 °C for three days under either anaerobic or aerobic conditions as the standard method [49].

### 5.2. Identification of Bacteria

#### 5.2.1. Isolation of Genomic DNA

Solitary colonies of bacterial cultures were isolated from the pour plates and inoculated in liquid nutrient broth (Scharlab, Barcelona, Spain). Following the DNA extraction protocol based on Wilson [50], 200 μL aliquots of 16 h cultures were mixed with a 1000 μL CTAB (Biochemica, Darmstadt, Germany) lysis buffer {2% (*w/v*) cetrimonium bromide (CTAB), 1.4 M NaCl, 100 mM Tris/HCl, 20 mM EDTA, pH 8.0} (Applichem Ltd. ITW Company, Darmstadt, Germany). The samples were incubated for 30 min at 65 °C and then put in 2 mL Lysing Matrix B tubes (MP Biomedicals Germany GmbH, Schwegel, Germany). Bead-based lysis was carried out for 20 s at 6500 rpm with Precellys 24 homogenizer (Peqlab Biotechnologie Ltd., Erlangen, Germany). Cell debris of lysed bacteria was separated by centrifugation (10 min at 14,000 rpm). To each 600 μL aliquot of the supernatant was added 240 μL of chloroform, stirred for 30 s, and centrifuged (20 min at 14,000 rpm). Aliquots (400 μL each) of the upper aqueous phase were mixed with equal volumes of isopropanol and were centrifuged again (10 min, 14,000 rpm). After discarding the supernatants, the pellets were washed with 500 μL aliquots of 70% ethanol, re-centrifuged, and left to dry at room temperature. Genomic DNA was re-suspended in 500 μL of water and was purified further using an Amicon Ultra filtration kit (Merck Millipore, Darmstadt, Germany) following the manufacturer’s instructions.

#### 5.2.2. PCR Method

In all PCR, the iProof high-fidelity PCR kit (BIO-RAD Ltd., Hercules, CA, USA Lithuania) was used with 27F (5′-AGAGTTTGATCCTGGCTCAG-3′) and 1492R (5′-TACGGTTACCTTGTTACGACTT-3′) primers of 16S rRNA [51]. PCR was performed at 98 °C for 3 min; after that, 98 °C for 30 s, 54 °C for 30 s, 72 °C for 45 s, for 30 cycles, and 72 °C for 7 min on T100 thermal cycler (BIO-RAD Ltd., Lithuania).

DNA amplicons were cleaned in a 0.8% agarose gel (BIOLINE), Nucleo SpinGel, and PCR clean-up column (Macherey-Nagel, Düren, Germany) kit to the protocols of the manufacturers. The PCR products were sequenced by BIOMI Ltd. (Gödöllő, Hungary). The sequences were submitted to the National Library of Medicine at the National Center for Biotechnological Information (NCBI) under accession numbers OP183257-OP183263.

### 5.3. Bacterial Cell Fractions

Bacterium strains were grown in nutrient broth (Scharlab; 16 h at 30 °C), and exponential phase cultures were centrifuged (8000 rpm, 10 min, 4 °C), the supernatants were removed, and the cell pellets were washed three times with 200 μL aliquots of sterile phosphate-buffered saline solution (PBS). After washing, the aliquots of the pellets were exposed to various chemicals to get different cell wall fractions based on Niederkorn et al. [31] and Goh et al. [52]: H_2_O (100 °C, 15 min), cell debris; 2% (*w*/*v*) sodium dodecyl sulphate (SDS) (100 °C, 15 min), purified cell wall fraction; 0.1 M HCl (100 °C, 15 min), teichoic acid fraction; 10% (*w*/*v*) trichloroacetic acid (TCA) (100 °C, 15 min), peptidoglycan fraction and 1M LiCl (100 °C, 15 min), S-layer proteins. After treatments, samples were centrifuged at 14,000 rpm 10 min, the supernatants were discarded, and the pellets were washed with 3 × 200 μL PBS. Bacterial cells and cell wall fractions were stored at −18 °C.

### 5.4. Mycotoxin Elimination

Deoxynivalenol (DON), zearalenone (ZEA), and aflatoxin B1 (AFB1) (Biopure, Romer Labs, Tulln, Austria) calibration solutions were purchased and used in appropriate dilution for mycotoxin elimination tests. Mycotoxins were diluted to the final concentration in phosphate-buffered saline (PBS) and were added at the following concentrations to living bacterial cells and different bacterial cell wall preparations: AFB1—24 μg/L, DON—700 μg/L, ZEA—100 μg/L. The toxin concentrations applied were based on literature search, the average toxin content of the forage samples, and preliminary mycotoxin tolerance studies [48]. All mycotoxin-supplemented samples were incubated in PBS for 1 h at 25 °C with shaking (250 rpm), centrifuged (8000 rpm, 10 min, 4 °C), and the supernatants were removed, extracted, and analyzed by HPLC. All assays were performed in triplicate, and positive controls (without cells or cell wall fractions) and negative controls (without mycotoxin) were included. HPLC detection of DON was carried out on Hitachi Elit LaChrom HPLC (San Jose, CA, USA) equipment. For deoxynivalenol (DON) measurement, the filtrated supernatant samples were loaded onto a Phenomenex (Torrance, CA, USA) RP-C18 column (125 × 4 mm, 5 µm) and detected with a diode array detector in UV 218 nm with acetonitrile: water (10:90) eluent.

For AFB1, the sample and methanol were mixed in 1:1 ratio and vortexed at high speed. HPLC detection of the tested mycotoxins was carried out in Dionex Ultimate 3000 (Thermo Scientific, Waltham, MA, USA) equipment [48]. The diluted extract was filtered and loaded onto a Phenomenex (Torrance, CA, USA) RP-C18 column (150 × 4.6 mm, 5 µm) with a Romer UV derivatization unit (Romer Labs Ltd., Tulln, Austria) and a fluorescence detector (ex360 nm, em440 nm) with methanol: water (45:55) eluent.

In samples exposed to ZEA, the mycotoxin contents of the supernatants and the pellets were also measured. Supernatants were treated with methanol in a 1:1 ratio and vortexed at high speed. Pellet-adsorbed ZEA was extracted by a mixture of acetonitrile–water–methanol (46:46:8) based on ZearalaTest^TM^ (VICAM, Watertown, USA). HPLC detection of the tested mycotoxins was carried out in Dionex Ultimate 3000 (Thermo Scientific) equipment [48]. The extracts were filtered and loaded onto a Phenomenex (Torrance, CA, USA) RP-C18 column (150 × 4.6 mm, 5µm) and a fluorescence detector (ex274 nm, em440 nm) with acetonitrile–water–methanol (46:46:8) eluent.

For AFB1, DON, and ZEA, the limits of detection (LOD) were 0.02 µg/L, 0.05 mg/L, and 2.6 µg/L, respectively. The retention times were 10.3 min, 3.2 min, and 8.9 min, respectively. The coefficient of variation within test repetitions was calculated and found to be under 15% in all cases.

### 5.5. Esterase Activity

Enzyme activity was measured spectrophotometrically based on the method of Castilo et al. [53]. The reaction mixture contained 800 µL 50 mM Tris-HCl buffer, pH 7.5, 100 µL *p*-nitrophenyl butyrate (Sigma-Aldrich, Saint Louis, USA) as substrate (8.1 mM in acetone), and 100 µL lysed (Precellys 24 homogenizer, Peqlab Biotechnologie Ltd., Erlangen, Germany) and PBS-washed cell debris or the heat-treated cell wall fraction samples. Enzyme activity was detected as *p*-nitrophenol deliberated after 10 min at 37 °C incubation (λ = 346 nm). The esterase activity was expressed as mM *p*-nitrophenyl released per min.

### 5.6. Statistical Analysis

Data analyses were done in Windows Excel Analysis ToolPac (Microsoft), where a t-probe (at *p* ≤ 0.05) was performed for the significance analysis. A correlation (Pearson) test of esterase activity and mycotoxin elimination was also done in Windows Excel data Analysis ToolPac (Microsoft).

## Figures and Tables

**Table 1:** Bacterium identification was based on 16S rRNA gene sequences. The sequences were submitted to the National Library of Medicine at National Center for Biotechnological Information (NCBI) under accession numbers OP183257-OP183263.

Description	Strain	Primer	Query Length	Homology	References
*Rummeliibacillus suwonensis*	AMK9/2	27F	1214	97.42%	[27]
	AMK9/2	1492R	1148	99.29%	
*Bacillus thuringiensis*	AMK10/1	1492R	1152	99.20%	[28]
*Lysinibacillus boronitolerans*	AMK9/1	27F	1165	98.37%	[29]
	AMK9/1	1492R	1156	98.93%	
*Lysinibacillus fusiformis*	AMK10/2	27F	1191	97.52%	[29]
	AMK10/2	1492R	1153	97.85%	

Source and homology search: NCBI database [30].

## Data Availability

Not applicable.
